# Knowledge, attitude and practice regarding insomnia prevention and treatment among medical students in Inner Mongolia

**DOI:** 10.3389/fpubh.2026.1685297

**Published:** 2026-03-11

**Authors:** Shirong Liu

**Affiliations:** Department of Traditional Chinese Medicine, Baotou Medical College, Inner Mongolia University of Science and Technology, Baotou, China

**Keywords:** attitudes, cross-sectional studies, health knowledge, Inner Mongolia, medical – psychology, practices, sleep initiation and maintenance disorders, students

## Abstract

This study aimed to assess the knowledge, attitude and practice (KAP) toward insomnia prevention and treatment among medical students in Inner Mongolia. A cross-sectional study was conducted at Baotou Medical College, Inner Mongolia University of Science and Technology from March 25, 2024 to June 17, 2024, using a self-designed KAP questionnaire. A total of 517 valid questionnaires were collected. Among the respondents, 273 (52.80%) were freshmen, 324 (62.67%) were female, and 112 (21.66%) had subclinical insomnia. The mean scores for knowledge, attitude, and practice were 13.09 ± 2.56 (adequate knowledge >14), 64.99 ± 10.74 (positive attitude >56), and 31.83 ± 8.94 (proactive practice > 31.5), respectively. The correlation analysis indicated a significant positive relationship between knowledge and attitude (*r* = 0.152, *p* < 0.001). Additionally, there was a correlation between attitude and practice (*r* = 0.333, *p* < 0.001). Multivariate logistic regression revealed that a higher attitude score (OR = 1.055, *p* < 0.001) and being in a relationship or married (OR = 1.712, *p* = 0.014) were associated with better practice, while received treatment for insomnia (OR = 0.095, *p* = 0.024) and clinical insomnia (OR = 0.523, *p* = 0.006) were negatively associated with practice. Medical students in Inner Mongolia exhibited insufficient knowledge and, while their attitudes were generally positive, students with symptoms demonstrated inadequate practice toward insomnia prevention and treatment. In this cross-sectional study, these findings suggest the need for enhanced screening and targeted interventions to help reduce the potential long-term harm of insomnia.

## Introduction

Insomnia is a major public health concern characterized by difficulties in initiating or maintaining sleep and impaired sleep quality, often leading to daytime dysfunction, fatigue, depression, and anxiety (1–3). Globally, insomnia affects approximately 10–20% of the population (4), while in China, nearly one-fifth of adults experience insomnia (5, 6). Among college students, the prevalence is substantially higher, largely attributed to lifestyle factors and academic stress (7, 8). Chronic insomnia is associated with poorer mental health outcomes, including an increased risk of depression (9–12), and is also associated with a higher prevalence of chronic conditions such as diabetes, obesity, and mood disorders (13–15), as well as comorbidity with other physical and mental health conditions (16).

Medical students, in particular, experience higher levels of stress compared to their non-medical peers, which contributes to insomnia and negatively impacts their quality of life (17). Given that they represent the future healthcare workforce, addressing insomnia in this group is critical. In particular, medical students in Inner Mongolia may face unique environmental and educational challenges compared with students in other regions of China. Inner Mongolia is characterized by a colder climate, large temperature fluctuations, and distinct dietary patterns, which may influence sleep habits and circadian rhythms. In addition, medical colleges in this region often adopt intensive training schedules and centralized campus management, potentially increasing academic workload and psychological stress. However, empirical evidence focusing on insomnia-related knowledge, attitudes, and practices among medical students in this region remains limited, highlighting the need for region-specific investigation. This study aims to fill gaps in the literature by focusing on insomnia prevalence and management among medical students, highlighting the urgent need for targeted interventions to improve their well-being and long-term professional performance.

The Knowledge-Attitude-Practice (KAP) model is pivotal in shaping health-related behaviors (18). The KAP framework, often assessed through a KAP questionnaire, is widely used to evaluate the knowledge, attitude and practice of target populations within the healthcare field, as well as to gage their demand and acceptance of relevant health information (19). This model is rooted in the idea that increased knowledge fosters more positive attitude, which in turn drive improved practice (20). Although the KAP framework relies on self-reported data and may be prone to social desirability bias, it remains a predominant tool in public health research. Its enduring value lies in its ability to systematically quantify the gaps between theoretical knowledge and actual behaviors, thereby providing a solid evidence base for designing targeted educational interventions.

This study aims to investigate insomnia prevalence and related knowledge, attitudes, and practices among medical students in Inner Mongolia, providing evidence to inform targeted educational and management strategies for insomnia prevention and intervention in this population.

## Materials and methods

### Study design and participants

This cross-sectional study was conducted at Baotou Medical College, Inner Mongolia University of Science and Technology, from March 25, 2024, to June 17, 2024, involving medical student participants. The study was approved by the Medical Ethics Committee of Baotou Medical College (2023[27]), and informed consent was obtained electronically prior to the administration of the questionnaire (only participants who consented were able to complete the survey).

Inclusion criteria:

1 Medical students at Baotou Medical College, Inner Mongolia University of Science and Technology;
2 Individuals who provided informed consent and voluntarily agreed to participate.
3 Participants who had the cognitive ability to complete research questionnaires and evaluations.

Exclusion criteria:

1 Students not currently enrolled (e.g., on leave of absence);
2 Individuals under 18 years of age;
3 Participants who withdrew or refused to complete the survey midway;
4 Questionnaires suspected of containing careless responses, such as those with logical inconsistencies or completed in less than 90 s or more than 1,200 s.

Medical students were conveniently sampled from Baotou Medical College, Inner Mongolia University of Science and Technology. Recruitment was conducted at designated locations within the college until the required sample size was achieved. The online survey was created using the WeChat mini-program “Questionnaire Star,” and distributed via a generated QR code. The QR code was shared at designated locations, allowing participants to access the questionnaire by scanning it. To ensure the quality and completeness of responses, each IP address was restricted to one submission, and all questionnaire items were mandatory. The research team reviewed all submitted questionnaires for completeness, internal consistency, and logical accuracy.

### Sample size calculation

The sample size was calculated using the following formula: *N* = (Z _(1-*α*/2)_/*δ*)^2^ × p × (1 - p) where N represents the required sample size, and p was set to 0.5 to ensure the maximum sample size. The significance level (*α*), also known as the type I error, was set to 0.05, with Z _(1-α/2)_ = 1.96. The standard error (*δ*) was assumed to be 0.05. Considering an anticipated effective questionnaire recovery rate of 90%, the final target was to collect at least 430 completed questionnaires.

### Questionnaire introduction

The initial draft of the questionnaire was developed and underwent two rounds of small-scale pilot testing (N1 = 51, N2 = 60). Based on the feedback from these pilot tests, the questionnaire was continuously refined. After the second pilot test, the overall Cronbach’s *α* coefficient for the questionnaire was 0.906, with the knowledge, attitude, and practice sections scoring 0.765, 0.948, and 0.926, respectively, indicating good internal consistency across all sections.

The questionnaire was developed based on previously published literature and established KAP survey frameworks related to sleep disorders and insomnia prevention and treatment (21). The final version includes four sections (Appendix): Demographic Information, Medical History, and Treatment History; Knowledge Dimension; Attitude Dimension; and Practice Dimension. The ISI score is interpreted as follows: 0–7 points indicates “no clinically significant insomnia,” 8–14 points suggests “subclinical insomnia,” 15–21 points indicates “clinical insomnia (moderate),” and 22–28 points indicates “clinical insomnia (severe).” The knowledge dimension consists of 18 questions, including both single-choice and multiple-choice formats. For single-choice questions, 1 point was awarded for each correct answer, and 0 point for incorrect answers. For the two multiple-choice questions, 2 points were awarded for all correct answers, 1 point for partially correct answers, and 0 point for incorrect answers. The total possible score for this section ranges from 0 to 20. The knowledge dimension covers core aspects of insomnia, including its definition, classification, and risk factors, and assesses participants’ understanding of sleep hygiene education, psychological and behavioral therapies (such as CBT-I), and general principles of commonly used insomnia treatments. Higher scores indicate a more comprehensive understanding of insomnia prevention and management. The attitude dimension uses a 5-point Likert scale and includes 16 questions, with a total score range of 16–80. The attitude dimension evaluates participants’ perceptions of major insomnia-related factors, such as academic pressure, psychological traits, and lifestyle habits, as well as their recognition of the importance of standardized insomnia treatment and key therapeutic goals, including improving daytime functioning. Higher scores reflect more positive attitudes toward insomnia prevention and treatment. The practice dimension is also based on a 5-point Likert scale and includes 9 questions, with a total score range of 9–45. The practice dimension assesses the frequency with which participants establish healthy sleep habits, correct misconceptions about sleep, and engage in physical exercise, as well as their preferences and recommendations regarding Western medicine, traditional Chinese medicine, and physical therapies when facing insomnia symptoms. Higher scores represent more proactive insomnia-related practices. Scores exceeding 14, 56, and 31.5, which correspond to 70% of the maximum possible scores in the knowledge, attitude, and practice sections respectively, indicate adequate knowledge, a positive attitude, and proactive practice (22, 23). This cutoff threshold was adopted based on previous KAP-related studies and common academic grading conventions, in which a score of 70% is widely regarded as a benchmark for satisfactory competency.

### Statistical methods

Descriptive analysis was performed on the demographic data and KAP scores of the respondents. Continuous variables were presented as means and standard deviations (Mean ± SD), while categorical variables were expressed as frequencies and percentages (n, %). To compare differences in knowledge (K), attitude (A) and practice (P) scores among respondents with different demographic characteristics, the Mann–Whitney U test was used for two-group comparisons, and the Kruskal-Wallis H test was applied for comparisons across three or more groups, as the data were non-normally distributed. Spearman’s rank correlation was used to analyze the relationships among knowledge, attitude, and practice scores. Furthermore, binary logistic regression analysis was conducted with the practice (P) score as the dependent variable to examine whether demographic characteristics served as risk factors for practice-related outcomes. Practice scores were dichotomized at a cutoff of 70% of the maximum score range to serve as the dependent variable. All statistical tests were two-sided, and a *p*-value less than 0.05 was considered statistically significant. SPSS 27.0 (IBM, Armonk, NY, United States) was used for all analyses.

## Results

### Demographic characteristics of participants

A total of 537 questionnaires were initially collected in this study. The following cases were excluded: (1) 7 participants who disagreed with the study; (2) 9 participants whose response time was less than 90 s or more than 1,200 s; (3) 2 participants who were under 18 years old; and (4) 2 participants majoring in computer science. This resulted in 517 valid questionnaires, yielding a validity rate of 96.28%. The overall Cronbach’s *α* coefficient for the final questionnaire was 0.899, with the knowledge, attitude, and practice sections scoring 0.687, 0.927, and 0.908, respectively. The KMO value was 0.892, indicating acceptable internal consistency.

The mean age of the 517 participants was 19.95 ± 1.51 years. Of these, 273 (52.80%) were freshmen, 324 (62.67%) were female, 294 (56.87%) reported a monthly household income of less than 8,000 yuan, 228 (44.10%) were only children, 389 (75.24%) were not in a relationship or were single, and 112 (21.66%) had subclinical insomnia (Table 1).

**Table 1:** Basic information of participants and KAP score.

Variables	N(%)	Knowledge, mean ± SD	*p*	Attitude, mean ± SD	*p*	Practice, mean ± SD	*p*
*N* = 517							
Total score		13.09 ± 2.56		64.99 ± 10.74		31.83 ± 8.94	
Academic year			0.549		0.116		0.178
Freshman	273 (52.80)	13.17 ± 2.43		64.08 ± 10.99		32.31 ± 8.76	
Sophomore	156 (30.17)	12.90 ± 2.74		65.56 ± 10.42		30.83 ± 9.20	
Junior and above	88 (17.02)	13.17 ± 2.61		66.82 ± 10.35		32.10 ± 8.99	
Major			0.195		0.353		0.280
Basic medical sciences	24 (4.64)	12.29 ± 2.93		64.83 ± 13.77		32.54 ± 8.35	
Preventive medicine	27 (5.22)	12.96 ± 2.12		62.56 ± 8.03		30.30 ± 8.46	
Clinical medicine	149 (28.82)	13.34 ± 2.67		65.16 ± 10.50		32.92 ± 8.69	
Medical technology	34 (6.58)	12.59 ± 2.46		64.18 ± 11.54		31.44 ± 9.62	
Dentistry	14 (2.71)	13.21 ± 2.67		65.43 ± 12.23		34.00 ± 8.81	
Traditional Chinese medicine	166 (32.11)	13.08 ± 2.66		65.48 ± 10.78		30.62 ± 9.41	
Nursing	57 (11.03)	13.46 ± 1.75		63.04 ± 11.24		32.84 ± 9.22	
Pharmacy	41 (7.93)	12.76 ± 2.79		67.95 ± 9.46		31.95 ± 7.86	
Other	5 (0.97)	11.80 ± 1.64		60.20 ± 5.07		28.20 ± 3.11	
Gender			0.010		0.346		0.163
Male	193 (37.33)	12.61 ± 3.01		64.29 ± 11.82		32.45 ± 8.54	
Female	324 (62.67)	13.37 ± 2.20		65.41 ± 10.05		31.46 ± 9.17	
Age	19.95 ± 1.51						
Ethnicity			0.080		0.143		0.691
Han	420 (81.24)	13.20 ± 2.45		65.42 ± 10.31		31.90 ± 8.93	
Minority	97 (18.76)	12.60 ± 2.92		63.16 ± 12.35		31.52 ± 9.06	
Residence			0.320		0.627		0.305
Urban or suburban	369 (71.37)	13.12 ± 2.60		65.09 ± 10.93		32.07 ± 8.74	
Rural	148 (28.63)	13.00 ± 2.47		64.75 ± 10.30		31.22 ± 9.43	
Monthly household income			0.169		0.378		0.353
<8,000	294 (56.87)	13.06 ± 2.44		64.43 ± 10.91		31.70 ± 9.23	
8,000–12,000	158 (30.56)	13.39 ± 2.34		65.73 ± 10.36		31.47 ± 8.48	
>12,000	65 (12.57)	12.48 ± 3.38		65.75 ± 10.93		33.26 ± 8.69	
Are you an only child			0.085		0.505		0.040
Yes	228 (44.10)	12.85 ± 2.69		65.25 ± 11.23		32.70 ± 8.96	
No	289 (55.90)	13.28 ± 2.44		64.79 ± 10.36		31.14 ± 8.89	
Relationship status			0.381		0.034		<0.001
No/single	389 (75.24)	13.12 ± 2.61		64.44 ± 10.66		31.03 ± 8.77	
In a relationship/married	128 (24.76)	12.99 ± 2.41		66.68 ± 10.85		34.27 ± 9.06	
Have you ever experienced any mental or psychological disorders?			0.109		0.283		0.384
No	498 (96.32)	13.06 ± 2.57		64.90 ± 10.75		31.89 ± 8.96	
Yes	19 (3.68)	13.84 ± 1.95		67.37 ± 10.66		30.11 ± 8.69	
Do you have any other medical conditions?			0.938		0.785		0.168
No	486 (94.00)	13.09 ± 2.56		65.03 ± 10.77		31.96 ± 8.98	
Yes	31 (6.00)	13.03 ± 2.54		64.35 ± 10.43		29.77 ± 8.18	
Have you ever received treatment for insomnia			0.838		0.475		<0.001
Yes	19 (3.68)	13.37 ± 2.31		67.26 ± 7.29		24.63 ± 5.39	
No	498 (96.32)	13.08 ± 2.57		64.91 ± 10.85		32.10 ± 8.94	
Severity of insomnia			0.432		0.739		<0.001
Insomnia of no clinical significance	390 (75.44)	13.08 ± 2.52		65.00 ± 11.24		32.71 ± 8.94	
Subclinical insomnia	112 (21.66)	13.15 ± 2.73		65.18 ± 9.08		29.41 ± 8.55	
Clinical insomnia (moderate)	15 (2.90)	12.87 ± 2.39		63.47 ± 9.34		27.07 ± 7.30	

### Knowledge, attitude, and practice scores

The mean knowledge score was 13.09 ± 2.56 (possible range: 0–20), which is lower than 70% of the total knowledge score (14), indicating that the participants did not achieve a good knowledge level. Demographic analyses showed that female participants had significantly higher knowledge scores compared to males (*p* = 0.010). Item-level analysis revealed notable gaps in participants’ understanding of insomnia symptoms, risk factors, and sleep hygiene knowledge, particularly regarding symptom recognition and appropriate pre-bedtime physical activity behaviors (Table 2).

**Table 2:** Knowledge dimension of the participants.

Items	N (%)		
	True	False	Not sure
1. Insomnia is a sleep disorder characterized by frequent and persistent difficulty in falling asleep and/or maintaining sleep, leading to dissatisfaction with sleep quality.	**451 (87.23)**	20 (3.87)	46 (8.9)
2. Difficulty falling asleep, difficulty returning to sleep after waking up, or frequent waking during sleep are symptoms of insomnia, but early morning awakening and frequent night wakings are not.	204 (39.46)	**263 (50.87)**	50 (9.67)
3. Risk factors for insomnia include:			
Age	**224 (43.33)**		
Gender	**113 (21.86)**		
Medical history	**208 (40.23)**		
Genetic factors	**169 (32.69)**		
Stress and life events	**232 (44.87)**		
Personality traits	**166 (32.11)**		
Response to environmental changes	**243 (47)**		
Mental factors	**234 (45.26)**		
Physical illness	**181 (35.01)**		
Medication	**184 (35.59)**		
All of above	**201 (38.88)**		
None of the above	7 (1.35)		
Not sure	15 (2.9)		
4. Insomnia can lead to decreased immunity, bodily dysfunction, and can cause a series of diseases such as gastrointestinal and cardiovascular issues.	**489 (94.58)**	4 (0.77)	24 (4.64)
5. Chronic insomnia, short-term insomnia, and other types of insomnia are usually differentiated by the course and frequency of the insomnia.	**466 (90.14)**	8 (1.55)	43 (8.32)
6. Compared to short-term insomnia, chronic insomnia is more commonly associated with identifiable triggering factors.	408 (78.92)	**48 (9.28)**	61 (11.8)
7. Some patients with short-term insomnia may develop chronic insomnia, requiring proactive and standardized treatment.	**464 (89.75)**	8 (1.55)	45 (8.7)
8. Regardless of the type of insomnia, the first step is always sleep hygiene education to prevent and correct poor sleep behaviors and beliefs.	**472 (91.3)**	11 (2.13)	34 (6.58)
9. The content of sleep hygiene education includes:			
9.1 Avoiding stimulants (such as coffee, strong tea, or smoking) several hours before bedtime (generally after 4 PM).	**485 (93.81)**	11 (2.13)	21 (4.06)
9.2 Avoiding alcohol before bedtime, as alcohol can interfere with sleep.	**413 (79.88)**	48 (9.28)	56 (10.83)
9.3 Engaging in regular physical exercise before bedtime, especially strenuous exercise, which can help improve sleep.	250 (48.36)	**226 (43.71)**	41 (7.93)
9.4 Eating heavily or consuming indigestible foods before bedtime.	185 (35.78)	**312 (60.35)**	20 (3.87)
9.5 Avoiding mentally stimulating activities or watching exciting books and TV shows at least an hour before bedtime.	**454 (87.81)**	37 (7.16)	26 (5.03)
9.6 Ensuring that the bedroom environment is quiet, comfortable, with appropriate light and temperature.	**501 (96.91)**	6 (1.16)	10 (1.93)
9.7 Maintaining a regular sleep schedule.	**500 (96.71)**	4 (0.77)	13 (2.51)
10. Medications commonly used to treat insomnia include:			
Benzodiazepine receptor agonists	**117 (22.63)**		
Benzodiazepines	**107 (20.7)**		
Melatonin receptor agonists	**175 (33.85)**		
Antidepressants	**69 (13.35)**		
Antiepileptic drugs	**37 (7.16)**		
Antipsychotics	**66 (12.77)**		
All of the above	**108 (20.89)**		
None of the above	9 (1.74)		
Not sure	172 (33.27)		
11. Psychological and behavioral therapy is the first-line treatment for insomnia, with CBT-I (Cognitive Behavioral Therapy for Insomnia) being the most common	**395 (76.4)**	7 (1.35)	115 (22.24)
12. The use of anti-insomnia medication should be individualized, adhering to principles of regular, low-dose, and continuous administration.	404 (78.14)	**24 (4.64)**	89 (17.21)

Bold is the correct option.

The mean attitude score was 64.99 ± 10.74 (possible range: 10–80), which is higher than 70% of the total attitude score (56), indicating that the participants have positive attitude. In the attitude dimension, most participants demonstrated a generally positive attitude toward the importance of sleep and the necessity of active insomnia treatment. However, participants showed relatively limited awareness of several specific sleep-related risk factors, indicating gaps in attitude toward comprehensive risk recognition (Table 3).

**Table 3:** Attitude dimension of the participants.

Items	N (%)				
	Strongly agree	Agree	Neutral	Disagree	Strongly disagree
Please assess your attitude toward the following factors affecting sleep. “Strongly agree” indicates a significant impact, while “Strongly disagree” indicates no impact at all					
1. High academic pressure	187 (36.17)	171 (33.08)	146 (28.24)	6 (1.16)	7 (1.35)
2. lack of psychological resilience	159 (30.75)	178 (34.43)	138 (26.69)	30 (5.8)	12 (2.32)
3. overly sensitive temperament	175 (33.85)	169 (32.69)	124 (23.98)	34 (6.58)	15 (2.9)
4. Neglecting work-life balance	168 (32.5)	190 (36.75)	117 (22.63)	30 (5.8)	12 (2.32)
5. Irregular lifestyle	206 (39.85)	188 (36.36)	82 (15.86)	31 (6)	10 (1.93)
6. Weak constitution or physical illness	157 (30.37)	184 (35.59)	112 (21.66)	43 (8.32)	21 (4.06)
7. snoring	131 (25.34)	113 (21.86)	175 (33.85)	65 (12.57)	33 (6.38)
8. Poor sleep environment	191 (36.94)	190 (36.75)	104 (20.12)	26 (5.03)	6 (1.16)
9. Drinking too much strong tea/milk tea/coffee before bed	166 (32.11)	155 (29.98)	87 (16.83)	64 (12.38)	45 (8.7)
10. Taking medications that may cause insomnia	169 (32.69)	158 (30.56)	82 (15.86)	53 (10.25)	55 (10.64)
Regarding insomnia, your attitude are:					
11. Insomnia should be taken seriously.	307 (59.38)	142 (27.47)	59 (11.41)	5 (0.97)	4 (0.77)
12. Insomnia requires proactive and standardized treatment.	310 (59.96)	142 (27.47)	57 (11.03)	7 (1.35)	1 (0.19)
Regarding the treatment of insomnia, you believe the focus should be:					
13. Increasing effective sleep time and/or improving sleep quality.	314 (60.74)	141 (27.27)	55 (10.64)	6 (1.16)	1 (0.19)
14. Improving insomnia-related daytime impairments.	294 (56.87)	160 (30.95)	55 (10.64)	7 (1.35)	1 (0.19)
15. Reducing or preventing the transition from short-term insomnia to chronic insomnia.	289 (55.9)	167 (32.3)	52 (10.06)	8 (1.55)	1 (0.19)
16. Reducing the risk of comorbid physical or mental disorders associated with insomnia.	304 (58.8)	155 (29.98)	52 (10.06)	4 (0.77)	2 (0.39)

The mean practice score was 31.83 ± 8.94 (possible range: 9–45), which is higher than 70% of the total practice score (31.5), indicating that the participants have proactive practice. Practice scores were significantly higher among students from single-child families (*p* = 0.040) and those who were in a relationship or married (*p* = 0.034 and *p* < 0.001). Practice scores showed a complex relationship with treatment for insomnia. While individuals who received treatment exhibited better knowledge and attitude, their practice scores were significantly lower compared to those who had not received treatment (*p* < 0.001). Item-level practice analysis indicated suboptimal adherence to several recommended behavioral strategies, particularly sleep restriction techniques, relaxation-based interventions, and regular physical exercise (Table 4).

**Table 4:** Practice dimension of the participants.

Items	N (%)				
	Always	Often	Sometimes	Occasionally	Never
For the prevention and control of insomnia, how often do you follow these practice?					
1. Establish good sleep habits and create a comfortable sleep environment.	240 (46.42)	138 (26.69)	115 (22.24)	20 (3.87)	4 (0.77)
2. Correct misconceptions about sleep and adopt positive, rational views on sleep.	265 (51.26)	123 (23.79)	104 (20.12)	22 (4.26)	3 (0.58)
3. Practice sleep restriction to improve nighttime sleep efficiency.	212 (41.01)	131 (25.34)	109 (21.08)	39 (7.54)	26 (5.03)
4. Listen to soft and soothing music to reduce sympathetic nervous system excitement, alleviate anxiety, and stress responses.	217 (41.97)	100 (19.34)	100 (19.34)	65 (12.57)	35 (6.77)
5. Engage in moderate to high-intensity aerobic exercise and moderate-intensity resistance training.	184 (35.59)	76 (14.7)	123 (23.79)	97 (18.76)	37 (7.16)
6. When you or your friends/family have symptoms of insomnia, how often do you choose or recommend hypnotherapy?	152 (29.4)	88 (17.02)	99 (19.15)	69 (13.35)	109 (21.08)
7. When you or your friends/family have symptoms of insomnia, how often do you choose or recommend taking melatonin or other Western medications?	132 (25.53)	55 (10.64)	120 (23.21)	78 (15.09)	132 (25.53)
8. When you or your friends/family have symptoms of insomnia, how often do you choose or recommend light therapy, electrotherapy, or other physical treatments?	118 (22.82)	50 (9.67)	83 (16.05)	58 (11.22)	208 (40.23)
9. When you or your friends/family have symptoms of insomnia, how often do you choose or recommend traditional Chinese medicine treatments?	154 (29.79)	102 (19.73)	153 (29.59)	57 (11.03)	51 (9.86)

### Correlation analysis

The correlation analysis indicated a significant positive relationship between knowledge and attitude (*r* = 0.152, *p* < 0.001). Additionally, there was a correlation between attitude and practice (*r* = 0.333, *p* < 0.001; Table 5).

**Table 5:** Correlation analysis of KAP scores.

Dimensions	Knowledge	Attitude	Practice
Knowledge	1		
Attitude	0.152 (*p* < 0.001)	1	
Practice	−0.033 (*p* = 0.449)	0.333 (*p* < 0.001)	1

### Multivariate analysis of practice dimensions

Multivariate logistic regression analysis highlighted that individuals with higher attitude scores (OR = 1.055, 95% CI: [1.035–1.074], *p* < 0.001) and those who are in a relationship or married (OR = 1.712, 95% CI: [1.116–2.626], *p* = 0.014) are more likely to exhibit positive practice behaviors. Conversely, individuals not received treatment for insomnia (OR = 0.095, 95% CI: [0.012–0.732], *p* = 0.024) and those with clinical insomnia (OR = 0.523, 95% CI: [0.328–0.833], *p* = 0.006) are less likely to have positive practice behaviors. These findings underscore the predictive value of these factors in determining practice outcomes. (Table 6).

**Table 6:** Factors of practice based univariable and multivariable logistic regression.

Practice	Univariate logistic regression		Multivariate logistic regression	
	OR (95%CI)	*p*	OR (95%CI)	*p*
Knowledge score	0.987 (0.922–1.058)	0.717		
Attitude score	1.055 (1.036–1.074)	**<0.001**	1.055 (1.035–1.074)	**<0.001**
Academic year				
Freshman	ref			
Sophomore	0.751 (0.498–1.132)	0.171		
Junior and above	0.919 (0.562–1.504)	0.736		
Major				
Clinical Medicine	1.338 (0.869–2.062)	0.186		
Traditional Chinese Medicine	0.954 (0.622–1.463)	0.828		
Other	ref			
Gender				
Male	1.076 (0.746–1.551)	0.697		
Female	ref			
Age	0.937 (0.830–1.057)	0.289		
Ethnicity				
Han	1.328 (0.833–2.116)	0.234		
Minority	ref			
Residence				
Urban or suburban	1.028 (0.694–1.522)	0.892		
Rural	ref			
Monthly household income				
<8,000	ref			
8,000–12,000	0.832 (0.555–1.246)	0.371		
>12,000	1.460 (0.851–2.507)	0.170		
Are you an only child				
Yes	1.378 (0.965–1.969)	0.078		
No	ref			
Relationship status				
No/single	ref		ref	
In a relationship/married	1.903 (1.270–2.852)	**0.002**	1.712 (1.116–2.626)	**0.014**
Do you have any other medical conditions?				
No	ref			
Yes	0.542 (0.238–1.236)	0.145		
Have you ever received treatment for insomnia				
No	0.085 (0.011–0.641)	**0.017**	0.095 (0.012–0.732)	**0.024**
Yes	ref		ref	
Severity of insomnia				
Insomnia of no clinical significance	ref		ref	
Clinical insomnia	0.479 (0.307–0.747)	**0.001**	0.523 (0.328–0.833)	**0.006**

The bold values indicate statistical significance (*p* < 0.05).

## Discussion

Overall, the findings indicate that medical students in Inner Mongolia demonstrated insufficient knowledge and selective awareness of insomnia-related risk factors, despite generally positive attitudes, while practical implementation of insomnia prevention and management behaviors remained suboptimal. These patterns highlight the need for targeted screening and intervention strategies.

Similar to our findings, an attitude study of dental students at a Middle Eastern university showed that dental students received a poor level of education in sleep medicine, resulting in insufficient knowledge in the field (24). Similarly, a study of Saudi undergraduate medical students showed a lack of knowledge about sleep and sleep disorders (25). Our research shows that the correlation analysis revealed a significant positive relationship between knowledge and attitude, and between attitude and practice. These findings are consistent with existing literature suggesting that improved knowledge is positively associated with attitude, which in turn correlates with better practice (20). It is noteworthy that although the correlations between the KAP dimensions were statistically significant, the effect sizes were relatively small, particularly for the association between knowledge and attitude or practice, reflecting the well-documented “knowledge–behavior gap” in health promotion research, whereby knowledge alone is often insufficient to translate into sustained behavioral change (26). In the specific context of medical education, structural and psychosocial factors such as high academic workload, persistent psychological stress, and constrained sleep environments may override theoretical knowledge, thereby preventing the translation of positive attitudes into consistent insomnia-related practices (27). Nevertheless, despite the small effect size, the positive association indicates that knowledge remains a necessary foundational component, even if it is insufficient by itself to drive meaningful behavioral change. These findings underscore the importance of multifaceted interventions that go beyond knowledge dissemination and address structural, psychological, and environmental barriers to behavior change. These findings are further supported by the multivariate logistic regression results, in which attitude scores were positively associated with practice scores, highlighting the close relationship between attitude and insomnia-related practice behaviors.

In this study, it was found that a significant proportion of students exhibited subclinical insomnia (21.66%) and clinical insomnia (2.90%), which is consistent with the reported prevalence of sleep disorders (28.9%) among college students in another study. Additionally, our study found that individuals with clinical insomnia had lower knowledge, attitude, and practice compared to asymptomatic students. Even those receiving treatment for insomnia showed better knowledge and attitude but still struggled with poor practice, likely due to the severity of their condition (28). This seemingly counterintuitive finding may be partly explained by confounding related to insomnia severity and treatment-seeking behavior. This counterintuitive finding may be explained by reverse causality: students with more severe or refractory insomnia are more likely to seek medical treatment, yet the severity of their condition may simultaneously make sustained adherence to behavioral recommendations more challenging (29). Furthermore, reliance on pharmacological interventions may inadvertently reduce motivation to engage in demanding behavioral modifications, such as sleep hygiene and lifestyle adjustment, which require continuous effort and self-regulation (30). Given the cross-sectional design of this study, the temporal sequence between treatment exposure and practice behaviors cannot be established, and this association should therefore be interpreted with caution. A non-randomized waitlist controlled study showed that a sleep psychology workshop for graduate students improved their knowledge, self-efficacy, preparedness, and confidence in managing insomnia, with lasting effects for 12 months after the workshop, and led to strong support for integrating sleep education and CBT-I into graduate psychology curricula (31). These results highlight the need for similar interventions aimed at undergraduate students to address the high prevalence of sleep disorders. In addition, CBT-I has been effective in strengthening the knowledge and practice of insomnia management (32). However, implementing full-scale CBT-I programs in university settings may be resource-intensive, requiring trained therapists and sustained institutional support. Therefore, stepped-care approaches and digital CBT-I platforms tailored for student populations may represent more feasible and scalable alternatives for medical colleges, allowing broader access while maintaining intervention effectiveness. Participants in a relationship or married, despite having lower knowledge scores, exhibited better attitude and practice in managing insomnia. This finding suggests that being in a supportive relationship may be associated with more favorable sleep-related behaviors, potentially due to shared routines, encouragement, or greater emotional stability (33).

The results indicate that while a majority of students correctly identified some general concepts about insomnia, there were notable gaps in their understanding of certain key aspects. Specifically, fewer than half of the participants demonstrated accurate recognition of insomnia symptoms and risk factors, with particularly low recognition rates for risk factors such as medical history, stress, and significant life events. Research has demonstrated that the consumption of sugar-sweetened coffee and energy drinks negatively impacts sleep quality, especially among adolescents. For instance, energy drink intake and sugar-sweetened coffee have been correlated with poorer sleep quality, with sugar-sweetened coffee affecting both sleep onset and maintenance, particularly among girls (34). Additionally, a systematic review found that caffeine consumption significantly reduces total sleep time and increases sleep onset latency, suggesting that coffee should be avoided at least 8.8 h before bedtime to mitigate these effects (35). The motivations behind caffeine consumption, such as alertness, habit, and socialization, further compound the issue as students may be unaware of the cumulative negative effects on their sleep quality (36). Moreover, our research revealed that students adopted a relatively passive approach to recognizing important risk factors, such as caffeinated beverages like coffee and tea. These findings highlight the need for educational programs to focus on these specific risk factors. Furthermore, our study revealed that while students generally demonstrated positive attitude toward insomnia prevention and treatment, with most strongly agreeing that sleep should be taken seriously (59.38%) and that insomnia requires active treatment (59.96%), there were notable gaps in their attitude toward specific risk factors (37). Fewer than one-third of students strongly agreed about the impact of factors such as psychological resilience (30.75%), weak constitution (30.37%), and snoring (25.34%) on sleep quality. This selective recognition of risk factors aligns with findings from Guo et al., who reported that medical students often underestimate the clinical significance of certain sleep disorders and their risk factors. The positive correlation between attitude and practice scores (*r* = 0.333, *p* < 0.001) in our study further emphasizes the importance of cultivating comprehensive attitude toward all aspects of sleep health. Educational interventions that specifically address these attitude gaps could significantly improve students’ overall approach to insomnia management. Previous research has shown that targeted educational programs can effectively improve attitude toward sleep disorders among healthcare students (38), suggesting that similar approaches could benefit medical students in Inner Mongolia. Institutions should consider implementing workshops and seminars that emphasize the long-term impact of insomnia, including the negative role of caffeine and stress, as well as the importance of early intervention. Peer-led discussion groups may also encourage students to share their perspectives on sleep hygiene, fostering a more proactive attitude. Regular assessments or self-reflection exercises could help students internalize the importance of addressing these risk factors in their daily routines (31).

While students practiced good sleep hygiene, there were notable gaps in other key areas, such as low adherence to regular physical exercise, limited use of music therapy, and a reluctance to recommend traditional treatments for insomnia. These deficiencies in practical implementation highlight the need for more focused training to improve students’ ability to apply comprehensive insomnia prevention and management strategies. These shortcomings suggest the need for targeted interventions to improve practical application (39, 40). To bridge this gap, practical interventions should be tailored to focus on the application of knowledge through hands-on training. For instance, students could be encouraged to create personalized sleep management plans that focus on evidence-based sleep hygiene, stimulus control strategies (e.g., leaving the bed if unable to fall asleep), and relaxation techniques, as well as receive feedback on their approaches to insomnia management. Although Sleep Restriction Therapy (SRT) is a core component of clinician-guided cognitive behavioral therapy for insomnia (CBT-I), clinical practice guidelines emphasize that it carries risks such as excessive daytime sleepiness and impaired reaction time, and therefore unguided self-administration is not recommended (41). Educational interventions for medical students should instead prioritize safe and self-manageable strategies, such as stimulus control and relaxation techniques, which have demonstrated effectiveness in low-intensity or self-help CBT-I programs (42). Consequently, educational interventions for medical students should prioritize safe and self-manageable behavioral modifications, whereas individuals with persistent or severe insomnia who may benefit from sleep restriction should be referred to qualified healthcare professionals for structured treatment. Additionally, collaborative efforts between medical institutions and healthcare providers could facilitate mentorship programs, allowing students to observe real-world insomnia treatment practice and assist healthcare professionals in providing patient care. These approaches are supported by prior studies, which have shown that experiential learning significantly improves the adoption of best practice in clinical environments. Lastly, institutions should promote awareness about the importance of integrating both Western medicine and traditional Chinese medicine approaches, ensuring students are equipped to recommend a holistic range of treatment options based on patient needs (43, 44).

This study has several limitations. First, the cross-sectional design limits the ability to establish causality between knowledge, attitude, and practice. Second, the use of self-reported questionnaires may introduce response bias, as participants might overestimate their knowledge and practice. Third, the study was conducted at a single medical college, which may limit the generalizability of the findings to other regions or institutions. Although the questionnaire was developed based on previously published literature and designed to comprehensively cover insomnia-related knowledge, attitudes, and practices, its content validity has not been formally evaluated using standardized psychometric validation procedures. Additionally, some subgroup analyses (such as only-child status and relationship status) may have been underpowered, and no formal adjustment for multiple comparisons was applied, which may increase the risk of type I error. Moreover, reliance on self-reported behaviors may have led to an overestimation of proactive practice due to social desirability bias. Finally, potential confounding factors, such as participants’ detailed mental health status (e.g., anxiety or depression levels), sleep environment characteristics, lifestyle habits, and academic workload intensity, were not controlled for in the regression analysis. Future studies should incorporate these variables to better isolate independent predictors of insomnia-related knowledge, attitudes, and practices.

## Conclusion

In conclusion, medical students in Inner Mongolia exhibited insufficient knowledge and, while generally positive, students with insomnia symptoms demonstrated inadequate practice toward prevention and treatment. This highlights the need for enhanced screening and targeted interventions to prevent the long-term impacts of insomnia. Specific measures should include: implementing regular sleep quality assessments using validated tools like the ISI as part of routine health checkups for medical students; developing peer support programs to promote positive sleep behaviors; integrating comprehensive sleep education modules into medical curricula with emphasis on both theoretical knowledge and practical management skills; establishing cognitive behavioral therapy for insomnia (CBT-I) workshops specifically tailored for medical students; and creating accessible sleep health resources through digital platforms.

## Data Availability

The original contributions presented in the study are included in the article/Supplementary material, further inquiries can be directed to the corresponding author.
