# Supplementary material for: Knowledge, attitude and practice regarding insomnia prevention and treatment among medical students in Inner Mongolia
Source: Front Public Health. 2026 Mar 11;14:1685297. doi: 10.3389/fpubh.2026.1685297 (PMC13013474; doi:10.3389/fpubh.2026.1685297)
Supplement: Supplementary file 1 [file Table_1.docx]

**Survey on the Knowledge, Attitudes, and Practices (KAP) of Insomnia Prevention and Treatment Among Medical Students in Inner Mongolia**

Dear Participant,

We sincerely invite you to participate in our research project. This study aims to investigate the knowledge, attitudes, and practices related to insomnia Prevention and Treatment among medical students in Inner Mongolia.

This survey is conducted anonymously, ensuring that your information will not be disclosed at any stage. The results of the survey will be used solely for academic research. Please feel free to complete the survey based on your actual situation.

We greatly appreciate your cooperation and support!

□ I acknowledge and agree that the collected data will be used for scientific research.

## Part 1 Basic Information

**1、You are a _______ medical student.**

a. undergraduate b. graduate

**2、Your academic year:_______（Options：**Freshman, Sophomore, Junior, Senior, Fifth Year, First-year Master's, Second-year Master's, Third-year Master's）

**3、Your major is in the following category：_______**（Options：Basic Medical Sciences, Preventive Medicine, Clinical Medicine, Medical Technology, Dentistry, Traditional Chinese Medicine, Nursing, Pharmacy, Other: _______）

**4、your gender：**

a.male b.female

**5、your age：**_______years old。

**6、ethnicity：**

a.han b.minority(______)

**7、Family residence：**

a. urban b. suburban c. rural

**8、Monthly household income：**

a.＜8000元 b.8000-12000 c.12000-20000 d.大于20000

**9、Are you an only child?**

a.yes b.no

**10、Have you had any study abroad experience?**

a.yes b.no

**11、Relationship status：**

a.single b. In a relationship (boyfriend) c. In a relationship (girlfriend) d. Married

**12、Have you ever experienced any mental or psychological disorders?**

a. No b. Yes, please specify: _________（e.g., Depression, Bipolar Disorder, Anxiety Disorder）

**13、Do you have any other medical conditions?**

a. No
b. Cerebrovascular disease
c. Cardiovascular disease
d. Endocrine disease
e. Kidney disease
f. Peripheral vascular disease
g. Osteoarticular disease
h. Other: _______

**14、Insomnia Severity Index (ISI) score:：。**

14.11 Have you had difficulty falling asleep recently (e.g., in the past two weeks)?

A. None
B. Mild
C. Moderate
D. Severe
E. Very severe

14.2Have you had difficulty staying asleep recently (e.g., in the past two weeks)?

A. None
B. Mild
C. Moderate
D. Severe
E. Very severe

14.3 Have you had early morning awakenings recently (e.g., in the past two weeks)?

A. None
B. Mild
C. Moderate
D. Severe
E. Very severe

14.4 How satisfied are you with your current sleep pattern?

A. Very satisfied
B. Satisfied
C. Neutral
D. Dissatisfied
E. Very dissatisfied

14.5To what extent do you think your sleep problem interferes with your daytime functioning (e.g., daytime fatigue, ability to handle work and daily tasks, attention, memory, mood)?

A. No interference
B. Slight
C. Some
D. Significant
E. Very significant

14.6Compared to others, to what extent do you think your insomnia affects or impairs your quality of life?

A. None
B. Slight
C. Some
D. Significant
E. Very significant

14.7How concerned/frustrated are you about your current sleep problem?

A. Not at all
B. Slightly
C. Somewhat
D. Quite a bit
E. Very much

**15、Have you ever received treatment for insomnia?**

a. No
b. Yes, with medication. Please specify: _______
c. Yes, with non-medication therapy. Please specify: _______

## part 2 Knowledge Dimension

**1、Insomnia is a sleep disorder characterized by frequent and persistent difficulty in falling asleep and/or maintaining sleep, leading to dissatisfaction with sleep quality.**

a. True b. False c. Not sure

**2、Difficulty falling asleep, difficulty returning to sleep after waking up, or frequent waking during sleep are symptoms of insomnia, but early morning awakening and frequent night wakings are not.**

a. True b. False c. Not sure

**3、Risk factors for insomnia include: (Multiple choice)**

a. Age
b. Gender
c. Medical history
d. Genetic factors
e. Stress and life events
f. Personality traits
g. Response to environmental changes
h. Mental factors
i. Physical illness
j. Medication
k. All of the above (Single choice only)
l. None of the above (Single choice only)
m. Not sure (Single choice only)

**4、Insomnia can lead to decreased immunity, bodily dysfunction, and can cause a series of diseases such as gastrointestinal and cardiovascular issues.**

a. True
b. False
c. Not sure

**5、Chronic insomnia, short-term insomnia, and other types of insomnia are usually differentiated by the course and frequency of the insomnia.**

a. True
b. False
c. Not sure

**6、Compared to short-term insomnia, chronic insomnia is more commonly associated with identifiable triggering factors.**

a. True
b. False
c. Not sure

**7、Some patients with short-term insomnia may develop chronic insomnia, requiring proactive and standardized treatment.**

a. True
b. False
c. Not sure

**8、Regardless of the type of insomnia, the first step is always sleep hygiene education to prevent and correct poor sleep behaviors and beliefs.**

a. True
b. False
c. Not sure

**9、The content of sleep hygiene education includes:**

**9.1** **Avoiding stimulants (such as coffee, strong tea, or smoking) several hours before bedtime (generally after 4 PM).**

a. True
b. False
c. Not sure

**9.2** **Avoiding alcohol before bedtime, as alcohol can interfere with sleep.**

a. True
b. False
c. Not sure

**9.3** **Engaging in regular physical exercise before bedtime, especially strenuous exercise, which can help improve sleep.**

a. True
b. False
c. Not sure

**9.4** **Eating heavily or consuming indigestible foods before bedtime.**

a. True
b. False
c. Not sure

**9.5** **Avoiding mentally stimulating activities or watching exciting books and TV shows at least an hour before bedtime.**

a. True
b. False
c. Not sure

**9.6** **Ensuring that the bedroom environment is quiet, comfortable, with appropriate light and temperature.**

a. True
b. False
c. Not sure

**9.7** **Maintaining a regular sleep schedule.**

a. True
b. False
c. Not sure

**10、Medications commonly used to treat insomnia include: (Multiple choice)**

a. Benzodiazepine receptor agonists
b. Benzodiazepines
c. Melatonin receptor agonists
d. Antidepressants
e. Antiepileptic drugs
f. Antipsychotics
g. All of the above (Single choice only)
h. None of the above (Single choice only)
i. Not sure (Single choice only)

**11、Psychological and behavioral therapy is the first-line treatment for insomnia, with CBT-I (Cognitive Behavioral Therapy for Insomnia) being the most common**

a. True
b. False
c. Not sure

**12、The use of anti-insomnia medication should be individualized, adhering to principles of regular, low-dose, and continuous administration.**

a. True
b. False
c. Not sure

## part 3 Attitude dimension

**Please assess your attitude towards the following factors affecting sleep. "Strongly agree" indicates a significant impact, while "Strongly disagree" indicates no impact at all:**

1、High academic pressure a. Strongly agree b. agree c. neutral d. disagree e. strongly disagree

2、Lack of psychological resilience a. Strongly agree b. agree c. neutral d. disagree e. strongly disagree

3、Overly sensitive temperament a. Strongly agree b. agree c. neutral d. disagree e. strongly disagree

4、Neglecting work-life balance a. Strongly agree b. agree c. neutral d. disagree e. strongly disagree

5、Irregular lifestyle a. Strongly agree b. agree c. neutral d. disagree e. strongly disagree

6、Weak constitution or physical illness a. Strongly agree b. agree c. neutral d. disagree e. strongly disagree

7、Snoring a. Strongly agree b. agree c. neutral d. disagree e. strongly disagree

8、Poor sleep environment a. Strongly agree b. agree c. neutral d. disagree e. strongly disagree

9、Drinking too much strong tea/milk tea/coffee before bed a. Strongly agree b. agree c. neutral d. disagree e. strongly disagree

10、Taking medications that may cause insomnia a. Strongly agree b. agree c. neutral d. disagree e. strongly disagree

**Regarding insomnia, your attitudes are:**

**11、Insomnia should be taken seriously.**

a. Strongly agree b. agree c. neutral d. disagree e. strongly disagree

**12、Insomnia requires proactive and standardized treatment.**

a. Strongly agree b. agree c. neutral d. disagree e. strongly disagree

**Regarding the treatment of insomnia, you believe the focus should be:**

**13、Increasing effective sleep time and/or improving sleep quality.**

a. Strongly agree b. agree c. neutral d. disagree e. strongly disagree

**14、Improving insomnia-related daytime impairments.**

a. Strongly agree b. agree c. neutral d. disagree e. strongly disagree

**15、Reducing or preventing the transition from short-term insomnia to chronic insomnia.**

a. Strongly agree b. agree c. neutral d. disagree e. strongly disagree

**16、Reducing the risk of comorbid physical or mental disorders associated with insomnia.**

a. Strongly agree b. agree c. neutral d. disagree e. strongly disagree

## Part 4 Practice Dimension

**For the prevention and control of insomnia, how often do you follow these practices?**

**1、Establish good sleep habits and create a comfortable sleep environment.**

a. Always
b. Often
c. Sometimes
d. Occasionally
e. Never

**2、Correct misconceptions about sleep and adopt positive, rational views on sleep.**

a. Always
b. Often
c. Sometimes
d. Occasionally
e. Never

**3、Practice sleep restriction to improve nighttime sleep efficiency.**

a. Always
b. Often
c. Sometimes
d. Occasionally
e. Never

**4、Listen to soft and soothing music to reduce sympathetic nervous system excitement, alleviate anxiety, and stress responses.**

a. Always
b. Often
c. Sometimes
d. Occasionally
e. Never

**5、Engage in moderate to high-intensity aerobic exercise and moderate-intensity resistance training.**

a. Always
b. Often
c. Sometimes
d. Occasionally
e. Never

**6、When you or your friends/family have symptoms of insomnia, how often do you choose or recommend hypnotherapy?**

a. Always
b. Often
c. Sometimes
d. Occasionally
e. Never

**7、When you or your friends/family have symptoms of insomnia, how often do you choose or recommend taking melatonin or other Western medications?**

a. Always
b. Often
c. Sometimes
d. Occasionally
e. Never

**8、When you or your friends/family have symptoms of insomnia, how often do you choose or recommend light therapy, electrotherapy, or other physical treatments?**

a. Always
b. Often
c. Sometimes
d. Occasionally
e. Never

**9、When you or your friends/family have symptoms of insomnia, how often do you choose or recommend traditional Chinese medicine treatments?**

a. Always
b. Often
c. Sometimes
d. Occasionally
e. Never
